# Comparison between liquid and tablet formulations in the treatment of congenital hypothyroidism

**DOI:** 10.1007/s12020-026-04663-y

**Published:** 2026-05-28

**Authors:** Anna Parzianello, Erika Cantarelli, Elisa Bortolamedi, Egidio Candela, Alessandra Cassio, Federico Baronio, Marcello Lanari, Rita Ortolano

**Affiliations:** 1 Pediatric Unit, IRCCS Azienda Ospedaliero-Universitaria di Bologna, Via Massarenti 11, Bologna, 40138 Italy; 2 Unit of Paediatrics, University Hospital of Parma, Parma, 43126 Italy; 3 Department of Medical and Surgical Sciences, Alma Mater Studiorum, University of Bologna, Via Massarenti 11, Bologna, 40138 Italy

**Keywords:** Congenital Hypothyroidism, Newborn Screening, Levothyroxine, LT4, Thyroid, Pediatric Endocrinology

## Abstract

**Aim:**

This study compares the efficacy and safety of three levothyroxine (LT4) formulations in pediatric patients with congenital hypothyroidism (CH).

**Methods:**

This is a retrospective-prospective observational monocentric study conducted at the Pediatric Unit, IRCCS Azienda Ospedaliero-Universitaria di Bologna, Italy. Patients’ data were collected from diagnostic confirmation following CH positivity on newborn screening (occurring between January 2019 and April 2024) through age 3 years. According to the LT4 pharmaceutical formulation used in routine clinical practice, patients were assigned to one of three specific groups: T (tablets), D (oral drops), or S (oral solution).

**Results:**

82 patients were enrolled (group D, *N* = 30; group S, *N* = 25; group T, *N* = 27). At 7–15 days of follow-up, the median TSH concentration was significantly higher in group S compared with group T (*p* = 0.020). Accordingly, the proportion of patients with serum TSH concentrations above the reference range was significantly higher in group S compared with group T at this time point (*p* = 0.005). Another difference was observed at 12 months of follow-up, when the proportion of patients with serum fT4 concentrations below the reference range was higher in group D than in groups S and T (*p* = 0.011). No treatment-related adverse effects were observed with any formulation.

**Conclusion:**

The three LT4 formulations were equally effective in normalizing and maintaining serum TSH and fT4 within reference ranges. However, the clinical relevance of the differences observed at specific time points requires further investigation.

## Introduction

Congenital hypothyroidism (CH) is the most common congenital endocrine disorder, defined by a deficiency of thyroid hormones at birth. Historically, it has been a major preventable cause of neurocognitive impairment in childhood, with cretinism representing its most severe manifestation [1]. This reflects the key role of thyroid hormones in central nervous system development from prenatal life to age 3 [2]. Since the early 1970s, newborn screening (NS) has enabled early diagnosis and treatment, improving outcomes and neurocognitive development in children [3].

Pharmacological treatment of CH consists of hormone replacement therapy with levothyroxine (LT4) [4], which is commercially available in various formulations. As this report only includes patients up to 3 years of age, formulations unsuitable for this age group were excluded. Therefore, the analysis focused on LT4 tablets (Eutirox^®^), oral drops (Tirosint^®^), and oral solution (Tifactor^®^), which were made commercially available at different times [5–7]. To date, no study has assessed all three LT4 formulations in pediatric CH patients up to 3 years, except for our preliminary data updated here [8].

According to the literature, previous studies in the pediatric CH population have only compared the efficacy of LT4 therapy between solid and liquid formulations [9–12] or between two liquid formulations [13, 14]. Larger studies are needed to optimise CH hormone replacement by tailoring LT4 dosing to formulation, especially early in treatment. This retro-prospective observational study compares the efficacy and safety of three LT4 formulations in pediatric CH, defined by normalization and maintenance of serum TSH and fT4 within reference ranges [4].

This monocentric study is conducted at the Pediatric Unit, Program of Endocrine-Metabolic Disorders of IRCCS Azienda Ospedaliero-Universitaria of Bologna, which includes the unique Regional Newborn Screening Center for Endocrine-Metabolic Disorders of the Emilia-Romagna region (Italy). Ethical approval was obtained from the Ethics Committee of IRCCS Azienda Ospedaliero-Universitaria di Bologna (012/2024/Oss/AOUBo).

## Materials and methods

This study included male and female patients born in Emilia-Romagna, Italy, between January 2019 and April 2024 who met the following inclusion criteria: positive result for CH at the NS test (TSH > 9 µU/ml); diagnostic CH confirmation (TSH > 10 µU/ml and/or fT4 below the lower limit of the age-specific normal range) with indication to start hormone replacement therapy; starting LT4 therapy according to guidelines [4] within 30 days of life exclusively in the formulations of tablets, oral drops, or oral solution; follow-up of at least 12 months without switching the LT4 pharmaceutical formulation; absence of complex syndromes and/or chromosomal abnormalities. According to the LT4 pharmaceutical formulation used in routine clinical practice, patients were assigned to one of the three specific groups: T (tablets), D (oral drops), or S (oral solution). Clinical and laboratory data were collected at diagnostic confirmation and during follow-up visits performed at 7–15 days after starting LT4 therapy, and at 1, 3, 6, 12, 24, and 36 months thereafter. To date, follow-up is ongoing for some patients.

## Results

82 patients were enrolled, all of whom were included in this statistical analysis (group D, *N* = 30; group S, *N* = 25; group T, *N* = 27). The median follow-up was 24 months for both groups D and S, and 36 months for group T. Upon diagnostic confirmation, the three groups were considered homogeneous with respect to patient characteristics [Table 1]. The LT4 dose (µg/kg/day), prescribed to patients and adjusted at each follow-up based on clinical assessment and thyroid function parameters, showed no statistically significant differences between the groups throughout the follow-up.

**Table 1:** Demographic, clinical and laboratory characteristics of patients at diagnostic confirmation, stratified by treatment group (D, S, T)

		D (*N* = 30)	S (*N* = 25)	T (*N* = 27)	*P* value
Sex	F, N(%)	17 (56.7)	13 (52.0)	18 (66.7)	*0.544*
	M, N(%)	13 (43.3)	12 (48.0)	9 (33.3)	
Family history of thyroid disease	N(%)	11 (36.7)	13 (54.2)	15 (55.6)	*0.283*
Comorbidities	N(%)	4 (13.3)	2 (8.0)	2 (7.4)	*0.707*
Maternal antibodies	N(%)	3 (10.3)	3 (12.5)	6 (23.1)	*0.383*
Dysgenesis	Yes, N(%) Thyroid in situ, N(%)	21 (70.0) 9 (30.0)	15 (60.0) 10 (40.0)	13 (48.1) 14 (51.9)	*0.244*
Thyroid in situ	Transient, N(%)	3 (33.3)	4 (40.0)	8 (57.2)	*0.274*
	Permanent, N(%)	5 (55.6)	2 (20.0)	3 (21.4)	
	n.a., N (%)	1 (11.1)	4 (40.0)	3 (21.4)	
Gestational age (weeks)	mean ± DS	39.52 ± 1.87	39.61 ± 1.42	39.67 ± 1.53	*0.935*
Neonatal Weight (SDS)	mean ± DS	-0.46 ± 1.13	0.11 ± 0.91	-0.52 ± 0.85	*0.158*
Birth length (SDS)	mean ± DS	-0.06 ± 0.99	0.14 ± 0.96	-0.16 ± 1.09	*0.216*
TSH at NS (µU/ml)	median (Q1-Q3)	94.60 (35.20–166.00)	68.60 (20.00-157.00)	26,40 (12.00-70.15)	*0.068*
TSH at diagnosis (µU/ml)	median (Q1-Q3)	201.25 (48.13–397.50)	118.95 (33.80-446.08)	79.90 (40.10-195.90)	*0.201*
fT4 at diagnosis (pg/ml)	median (Q1-Q3)	6.45 (3.05–7.45)	6.15 (3.73–10.13)	8.40 (4.75–11.45)	*0.288*
Time to therapy initiation (days)	mean ± DS	9.10 ± 4.10	9.24 ± 3.83	10.81 ± 5.62	*0.313*
LT4 dose (µg/kg/die)	mean ± DS	10.36 ± 1.34	9.34 ± 2.06	10.37 ± 2.43	*0.108*

Since the groups were homogeneous, clinical outcomes are reported below.

We observed comparable median serum TSH and fT4 concentrations among the three groups during follow-up visits. The only exception was observed 7–15 days after therapy initiation, when the median TSH concentration was significantly higher in group S than in group T [13.80 (4.50–28.50) µU/mL vs. 3.05 (1.20–10.33) µU/mL; *p* = 0.020], whereas group D showed a pattern more similar to group T, with no significant difference compared with group S. Nevertheless, when considering the percentage reduction in median serum TSH concentrations during the initial phase of treatment, no differences were observed between the groups at 7–15 days (*p* = 0.225) or at 1 month (*p* = 0.329) of follow-up.

**Fig. 1:**
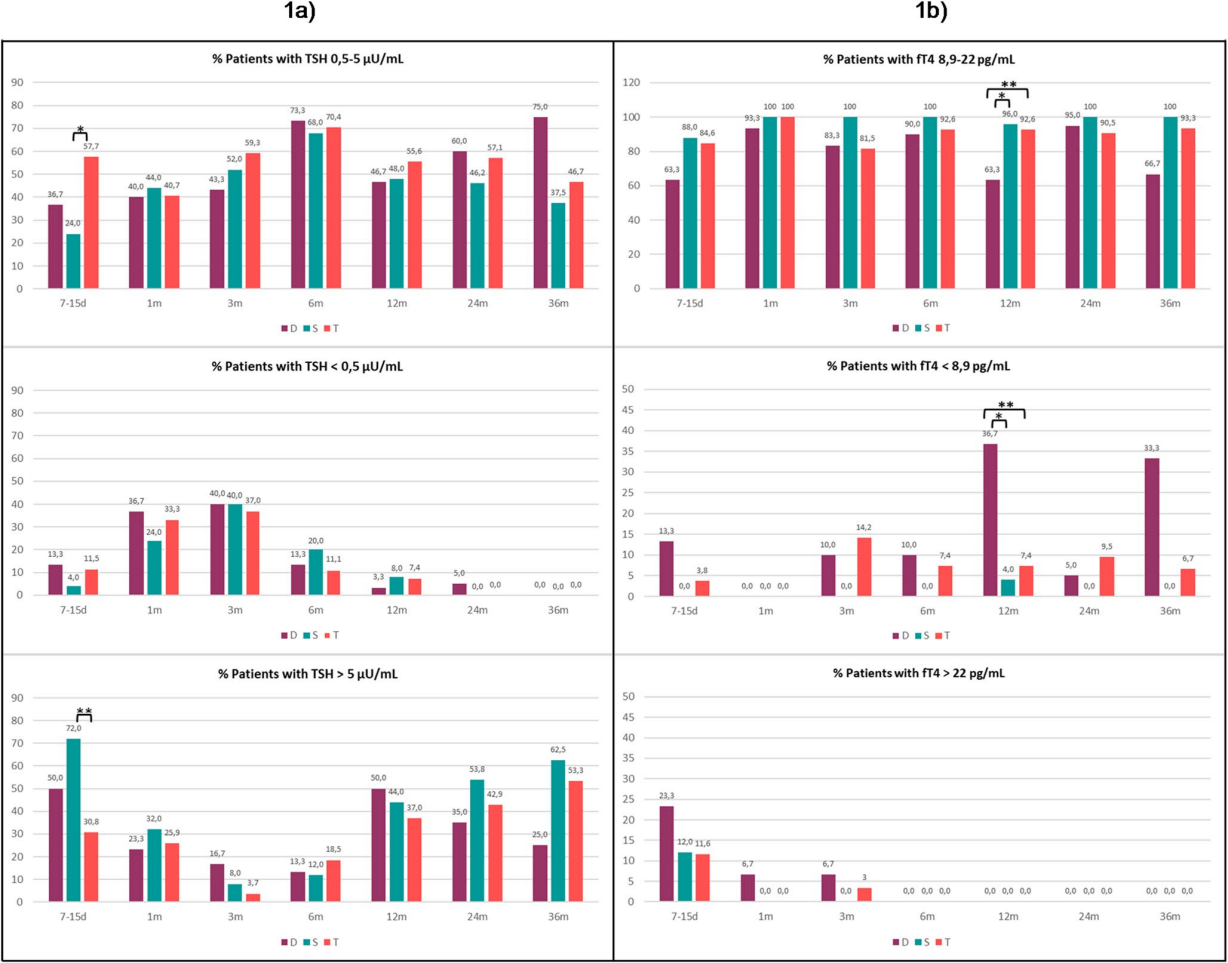
Proportions of patients in the three groups (D, S,T) with serum TSH (**a**) (*p = 0.023; **p = 0.005)] and fT4 (**b**) (*p = 0.004; **p = 0.011)] concentrations within, above, or below the reference range at the different evaluation timepoints

Subsequently, the proportions of patients with TSH and fT4 serum concentrations within, above, or below the reference ranges were assessed in the three groups at each follow-up [Fig. 1].

In agreement, at 7–15 days, the proportion of patients with serum TSH concentrations above the reference range was significantly higher in group S compared with group T [72.0% vs. 30.8%; *p* = 0.005]. (Fig. 1A). No statistically significant differences in the proportion of patients with FT4 in the normal range emerged, which was found in most patients of all groups already at this time point [63.3% (D), 88% (S), 84.6%(T)].

At 12 months, the proportion of patients with serum fT4 concentrations below the reference range was higher in group D compared with both group S and T [36.7%(D) vs. 4.0%(S), *p* = 0.004; 36.7%(D) vs. 7.4%(T), *p* = 0.011].

No further significant differences in the proportions of patients with TSH and fT4 serum concentrations within, above, or below the reference during follow-up were observed.

Finally, the annual rates of LT4 overtreatment and undertreatment were calculated for each year of follow-up, as described by *Ortolano et al.* [8]. No statistically significant differences were observed, except in the third year of follow-up, when the LT4 undertreatment rate was significantly higher in group S than in group D (68% vs. 29%, *p* = 0.035). No treatment-related adverse effects were observed with any formulation.

## Discussion

In our cohort, hormone replacement therapy proved to be equally effective in normalizing fT4 concentrations in CH patients treated with a comparable LT4 dose administered as tablets, oral drops, or oral solution. The trend of laboratory parameters across the three groups is consistent with our previously published preliminary data [8], thereby confirming those findings.

However, differences were observed in the timing of thyroid function control, not always consistent with findings reported in the pediatric literature as described below.

Patients receiving the oral solution showed a greater initial tendency toward undertreatment compared to other formulations, as reflected by both higher median serum TSH concentrations and a higher proportion of patients with TSH values above the reference range at 7–15 days from therapy start. This observation, consistent with our preliminary results [8], contrasts with *Tuli et al.* [13], who reported a significant initial tendency toward overtreatment with the oral solution, despite a similar LT4 dose used. Moreover, the tendency toward undertreatment with the oral solution observed in our cohort appears to recur during the maintenance phase, as reflected in the third annual undertreatment rate.

The higher undertreatment rate observed in the group treated with oral solution may be partially explained by the lower LT4 doses used at diagnosis compared to drops and tablets, although this difference was not statistically significant and was not observed at subsequent time points. This finding could be related to the monocentric design of the study and our local clinical practice; therefore, a multicentric study is indicated to reduce this bias and verify the reproducibility of our results.

Moreover, comparisons with the literature are limited, as follow-up in this formulation in other studies did not exceed six months, which limits the strength of this finding.

Notably, the tendency toward undertreatment with the oral solution is not reflected in serum fT4 concentrations in our cohort. In fact, we observed a similar normalization rate of fT4 as early as 7–15 days, and, better yet, only patients treated with this formulation consistently exhibited fT4 values within the normal range for nearly the entire follow-up. In contrast, *Vincenzi et al.* [14] reported significantly lower LT4 doses and significantly higher serum fT4 concentrations in patients treated with the oral solution, although fT4 levels remained within the normal range.

Unlike previous reports [9, 10, 12], we did not find a statistically significant major rate of overtreatment in group D at the first follow-up visits. In contrast, in our cohort, we observed a tendency toward undertreatment rather than overtreatment among patients treated with oral drops, as evidenced by the higher proportion of patients with serum FT4 concentrations below the reference range at 12 months. However, this may be explained by less frequent follow-up visits, which delayed dose adjustments following patient weight gain and, consequently, delayed optimization of clinical outcomes.

Finally, patients of our cohort treated with tablets overall showed lower rates of both overtreatment and undertreatment. However, *Vigone et al.* [12] observed significantly lower serum TSH concentrations in those treated with tablets compared with those treated with oral drops at the second year of follow-up, albeit within the reference range. To date, no studies other than *Vigone et al.*. have included a follow-up period exceeding 12 months to confirm these findings. Notably, LT4 doses are in the microgram range and are based on patients’ body weight, which is highly variable, particularly during the early stages of life [4]. Therefore, dose personalization is essential to ensure the effectiveness of hormone replacement therapy, especially given the narrow therapeutic index of LT4 [15]. Thus, accurate LT4 dosing represents a crucial aspect in the management of CH. In this context, the limited handling and dosing flexibility of LT4 tablet formulations are well recognized, whereas liquid formulations were specifically developed to overcome these limitations, thus improving compliance [16]. Conversely, real-world data from our cohort suggest that LT4 tablets result in more consistent dosing, with a lower risk of under- or overtreatment compared with liquid formulations.

Improving our knowledge of this topic may significantly contribute to better management of therapy in terms of the entity and timing of dose adjustments.

## Conclusion

This report directly compares the efficacy of three LT4 formulations currently authorized for CH pediatric patients (tablets, oral drops, and oral solution). The update of our previously published data [10] contributes to increasingly robust findings of our study, with the growing sample size and extended follow-up period as the major strengths. Nevertheless, direct comparison with the available literature is not always possible; therefore, the existing discrepancies between our findings and previous reports warrant in-depth investigation. Notably, further studies should be conducted to confirm whether differences in over- or undertreatment dosage exist among the three LT4 formulations and so we are conducting a multicentre Italian study with a larger population to confirm or not these differences and to evaluate their potential clinical implications, despite similar overall efficacy. 

## Data Availability

No datasets were generated or analysed during the current study.
